# The incidence of metabolic syndrome in psoriasis patients and its correlation with disease activity: a systematic review and meta-analysis

**DOI:** 10.3389/fmed.2025.1593003

**Published:** 2025-05-02

**Authors:** Zongyang Li, Zheng Gu, Jingyu Xiang, Xiaoyan Zhang

**Affiliations:** ^1^Graduate School, Beijing University of Chinese Medicine, Beijing, China; ^2^Department of Dermatology, China-Japan Friendship Hospital, Beijing, China

**Keywords:** psoriasis, metabolic syndrome, disease activity, meta, incidence

## Abstract

**Objective:**

To explore the association between psoriasis and metabolic syndrome (MetS) and analyze the impact of disease activity on the risk of MetS occurrence.

**Method:**

This systematic review and meta-analysis used computer searches to search for relevant literature on psoriasis and MetS in databases including China National Knowledge Infrastructure (CNKI), PubMed, Web of Science, Cochrane Central Register of Controlled Trials (CENTRAL) and Embase. The search period was from the establishment of the database to February 8, 2025. Inclusion in case–control, cohort studies and cross-sectional, with language restrictions in Chinese and English during retrieval. After independent screening of literature, extraction of data, and evaluation of risk bias for inclusion in the study by two evaluators, meta-analysis and subgroup analysis were conducted using Stata17.0 software.

**Result:**

A total of 12 studies were analyzed, encompassing 9,641 patients with psoriasis and 2,554 patients suffering from MetS alongside psoriasis. The incidence of metabolic syndrome in psoriasis patients was analyzed and the combined effect size was 26.49% [95% CI (25.61, 27.39%)]. Results from the meta-analysis indicated that, in comparison to the control group, psoriasis patients demonstrated a heightened risk of developing MetS [OR = 1.27, 95% CI (1.21–1.33), *p* < 0.001]. Subgroup analysis revealed that patients with severe psoriasis (PASI≥10) had a significantly increased risk of developing metabolic syndrome [OR = 2.25 95% CI (1.27, 3.99), *p* < 0.001], indicating that greater disease activity is associated with an elevated likelihood of MetS occurrence.

**Conclusion:**

Psoriasis is positively correlated with MetS risk, and increased disease activity further increases the risk. It is necessary to strengthen screening for metabolic abnormalities and multidisciplinary management.

## 1 Introduction

Psoriasis is an autoimmune skin disorder charaterized by chronic inflammation chronic inflammatory. Typically, the scaly red patches associated with psoriasis lesions are most prominent during the winter and spring, with some relief in summer and autumn (1). This condition, known as psoriasis, is a persistent systemic illness that usually presents with erythema, scaly skin and itchiness. It is not limited to the skin, but also involves other vital organs, forming a systemic chronic subclinical state.

The origins of psoriasis are multifaceted, influenced by various elements including genetics, infections, and environmental factors which contribute to its pathogenesis. At the same time, psoriasis patients produce a significant amount of inflammatory factors, such as IFN−γ (interferon γ ), IL-22 (interleukin-22), TNF−α (tumor necrosis factor α ) and IL−1β (interleukin-1 β ). The continuous and repeated release of these cytokines promotes the proliferation of keratinocytes (the predominant cells in the skin), which leads to the breakdown of skin barrier, the intensification of the inflammatory response, the dysregulation of the immune system and a cycle of long-term inflammation. The course of psoriasis is long and often volatile, and the repeated attacks of the disease make patients face great pressure on quality of life and physical and mental health. Psoriasis not only affects the skin, but also may lead to a series of comorbidities. Comorbidity refers to two or more diseases exist at the same time, and often affect each other, psoriasis and the occurrence of these comorbidity is closely related, some comorbidity may aggravate the symptoms of psoriasis, on the contrary, psoriasis itself will become the inducement of these comorbidity. Common psoriatic comorbidities include psoriatic joint disease, MetS, heart and vascular disorders, mood disorders, chronic inflammatory bowel disorders and nonalcoholic fatty liver disease. Recent studies have found a close association between it and MetS (2). Notably, emerging evidence highlights a robust bidirectional relationship between psoriasis and MetS, a clinical cluster characterized by central obesity, hypertension, insulin resistance, and dyslipidemia, which significantly elevates cardiovascular morbidity and mortality (3, 4). MetS includes hyperglycemia, hypertension, centripetal obesity and dyslipidemia. If MetS is not discovered and treated in time, it will cause great damage to the body and cause serious consequences. This can lead to subsequent cardiovascular disease (such as coronary heart disease, cerebrovascular events, coronary artery disease, stroke and heart failure), chronic kidney dysfunction (CKD), respiratory problems, and polycystic ovary syndrome (PCOS). Therefore, to recognize the association between psoriasis and MetS, and to identify the MetS group in psoriasis patients as early as possible will play an important role in the follow-up survival and physical recovery of patients. Additionally, the limitation of cross-sectional designs in such studies should be acknowledged, as they cannot fully establish causality or account for the temporal relationship between psoriasis and comorbid conditions. Language bias may also pose challenges in interpreting data from studies published in different regions or languages, which could potentially affect the generalizability of findings. Early recognition of MetS in psoriasis patients is critical for mitigating long-term health risks and improving clinical outcomes. Therefore, elucidating the relationship between the two can provide a basis for early clinical intervention and reduce the risk of comorbidities. This study quantified the risk of MetS occurrence in psoriasis patients through meta-analysis and explored the moderating effect of disease activity on risk.

## 2 Study population and protocol

### 2.1 Systematic literature retrieval

A systematic literature search was conducted across five electronic databases: CNKI, PubMed, Web of Science, CENTRAL and Embase, covering literature published from database inception to February 8, 2025. The search strategy aimed to identify studies exploring the investigating psoriasis-MetS comorbidity. For instance, the detailed PubMed search strategy included the following terms: (“Psoriasis” [Mesh] OR “psoriatic”) AND (“Metabolic Syndrome” [Mesh] OR “insulin resistance” OR “dyslipidemia”) AND (“prevalence” OR “incidence” OR “epidemiology”). To minimize selection bias, supplementary manual searches were performed by screening reference lists of eligible articles.

### 2.2 Eligibility assessment

Inclusion Criteria: (1) Study design: Observational studies including cross-sectional, case–control, or prospective/retrospective cohort designs. (2) Population: Case group comprising psoriasis patients; control group comprising non-psoriasis individuals. (3) Diagnostic criteria: Psoriasis diagnosis must be clinically or pathologically confirmed. Metabolic syndrome (MetS) must be diagnosed using either the NCEP-ATP III criteria or WHO criteria (5, 6). (4) Outcome: Reported prevalence of MetS. (5) Data accessibility: Availability of adjusted odds ratios (ORs) with 95% CIs, or primary data allowing OR/CI calculation. (6) Publication status: Peer-reviewed original articles.
Exclusion Criteria: (1) Duplicate publications. (2) Unclear diagnostic criteria for psoriasis or MetS. (3) Non-Chinese or non-English literature. (4) Studies with low quality (e.g., high risk of bias in methodology). (5) Incomplete or inaccessible data. (6) Studies lacking a control group; studies with flawed experimental design, data, or statistical methods. (7) Non-eligible study designs (e.g., reviews, case reports, or non-observational studies). (8) Non-target population (e.g., studies not focusing on psoriasis patients). (9) Irrelevant outcomes (e.g., no MetS prevalence or correlation data). (10) Unavailable data (e.g., insufficient raw data for analysis).

### 2.3 Data extraction

Two investigators independently reviewed eligible studies and abstracted the following parameters: (1) first author, publication year, geographic region, and study design; (2) diagnostic criteria and case counts of MetS; and (3) psoriasis-specific metrics, including Psoriasis Area and Severity Index (PASI) scores and disease duration. Inter-reviewer discrepancies were resolved through consensus with a senior researcher.

PASI assessments, performed by board-certified dermatologists, stratified psoriasis severity: scores <10 indicated mild-to-moderate disease, whereas scores ≥10 denoted severe manifestations.

The diagnosis of metabolic syndrome (MetS) in this study aligned with the National Cholesterol Education Program Adult Treatment Panel III (NCEP-ATP III) guidelines, which require fulfillment of ≥3 out of 5 pathophysiological components. These components reflect insulin resistance and cardiometabolic risk: (1) Dysglycemia: Fasting plasma glucose (FPG) ≥ 100 mg/dL (5.6 mmol/L ) or current glucose-lowering therapy. (2) Hypertension: Systolic/diastolic blood pressure ≥130/85 mmHg or documented use of antihypertensive agents. (3) Atherogenic dyslipidemia: Elevated triglycerides (TG): ≥150 mg/dL (1.7 mmol/L ) or lipid-modifying treatment. (4) Reduced high-density lipoprotein cholesterol (HDL-C): <40 mg/dL (1.0 mmol/L ) in males or <50 mg/dL (1.3 mmol/L ) in females. (5) Central adiposity: Waist circumference thresholds ≥102 cm (males) or ≥88 cm (females), adjusted for ethnicity where applicable. Notably, the International Diabetes Federation (IDF) and American Heart Association (AHA) definitions emphasize abdominal obesity as a prerequisite, whereas NCEP-ATP III adopts a non-hierarchical approach. To ensure consistency, studies employing alternative criteria (e.g., WHO 1999 requiring insulin resistance quantification) were excluded from the pooled analysis.

### 2.4 Methodological appraisal and risk of bias quantification

The Newcastle-Ottawa Scale (NOS) is widely employed for evaluating the methodological quality of cohort and case–control studies. The scale evaluates 3 aspects: the selection of study subjects, the comparability between groups, and the measurement of exposure or outcome, with a total score of 9 points. The NOS utilizes a total of 9 points, with scores ≥7 indicating high-quality studies, scores between 4 and 6 reflecting moderate quality, and scores ≤3 signifying low quality. The AHRQ observational study quality assessment tool, on the other hand, is specifically designed for evaluating bias risk in cross-sectional studies. This tool includes 11 items, with a total score of 11 points. A score of 0–3 indicates low quality, 4–7 points indicate moderate quality, and 8–11 points indicate high quality. Both tools are integral in ensuring that the results of observational research are credible and that the risk of bias is appropriately assessed.

### 2.5 Statistical analysis

Meta-analysis was conducted utilizing Stata 17.0 software. The odds ratio (OR) along with the 95% confidence interval (CI) were chosen as the primary outcome metrics for evaluating the effect size. The I2 statistic was employed to examine the level of heterogeneity across the studies included in the analysis. If I2≤50% , suggesting low heterogeneity, a fixed-effects model was applied; if I2>50% , indicating high heterogeneity, a random-effects model was used. Additionally, subgroup analysis was performed to explore potential variations based on the fundamental characteristics of the studies. To further ensure the robustness of the findings, a sensitivity analysis was conducted, which helped evaluate the consistency and reliability of the results across different scenarios.

## 3 Results

### 3.1 Literature search and evaluation results

**Figure 1:**
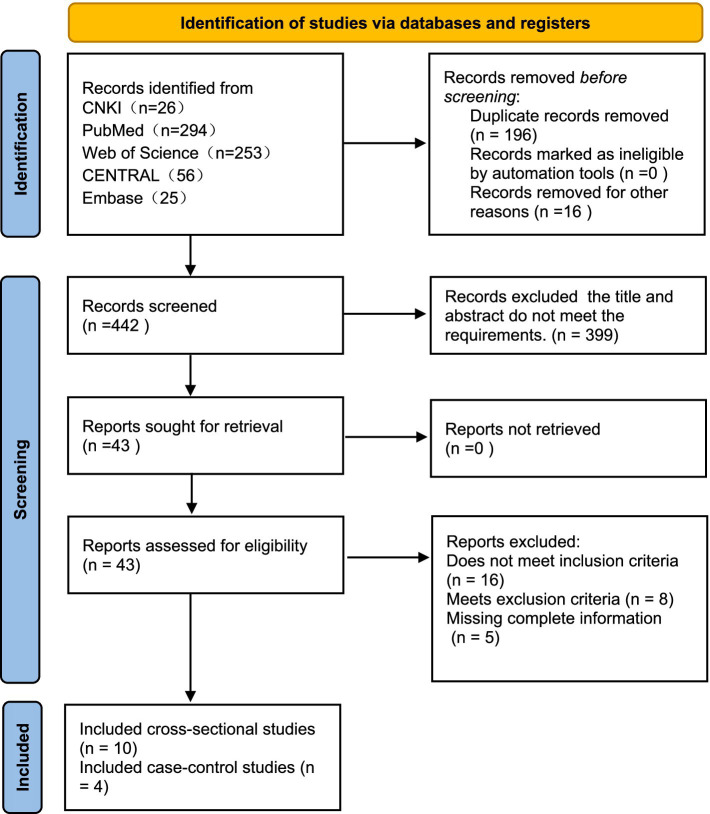
Study screening and selection flow.

A total of 654 articles were identified through the literature search, including 5 articles obtained from literature reviews. After initial screening and removal of duplicates, 442 articles remained. After assessing title and abstract eligibility, 399 publications were excluded for non-conformance with predefined inclusion criteria. After full-text review, an additional 29 articles were excluded. Ultimately, 14 articles were included in the meta-analysis, involving 9,641 psoriasis patients, with 2,554 cases of psoriasis combined with MetS (Figure 1). Among the 14 studies, all employed the revised NCEP-ATP III criteria for diagnosing MetS, consisting of 10 cross-sectional studies and 4 case–control studies. Investigations into the relationship between MetS and psoriasis were conducted in various geographic regions, including China, the United States, the Middle East, Denmark, India, and Italy. The meta-analysis of 14 studies showed no significant heterogeneity between studies ( I2=56% ). To account for the heterogeneity across studies, a random-effects model was utilized. In terms of study quality, 12 articles were classified as high quality, while 2 were of moderate quality. Detailed characteristics and bias risk assessments are presented in Table 1.

**Table 1:** Study population characteristics: psoriasis and metabolic syndrome.

Study	Study setting	Study design	Total no. of psoriasis		Total no. of control	
			With MetS	Without MetS	With MetS	Without MetS
Aalemi 2021 (14)	Afghanistan	Cross-sectional	42	72	24	90
Gui XY 2018 (15)	China	Cross-sectional	123	736	171	1,547
Itani S 2016 (16)	Lebanon	Cross-sectional	53	97	27	123
Parodi A 2014 (17)	Italy	Cross-sectional	97	263	55	305
Miller IM, 2014 (18)	Denmark	Cross-sectional	249	647	3,032	14,016
Kokpol C 2014 (19)	Thailand	Cross-sectional	98	101	61	138
Pehlevan 2013 (20)	Turkey	Case–control	21	38	12	70
Langan SM 2012 (21)	England	Cross-sectional	1,389	2,676	10,515	30,135
AL-Mutairi 2010 (22)	the Middle East	Case–control	265	1,396	124	1711
Takahashi H 2010 (23)	Japan	Cross-sectional	38	120	25	129
Nisa N 2010 (24)	India	Cross-sectional	42	108	9	141
Chen YJ 2008 (25)	Chia	Case–control	10	67	13	68
Gisondi P 2007 (26)	Italy	Case–control	102	210	69	265
Sommer DM 2006 (27)	–	Cross-sectional	25	556	11	1,033

“–” indicates that no specific information was provided in the included original studies.

### 3.2 Meta-analysis results

#### 3.2.1 The relationship between MetS and psoriasis

**Figure 2:**
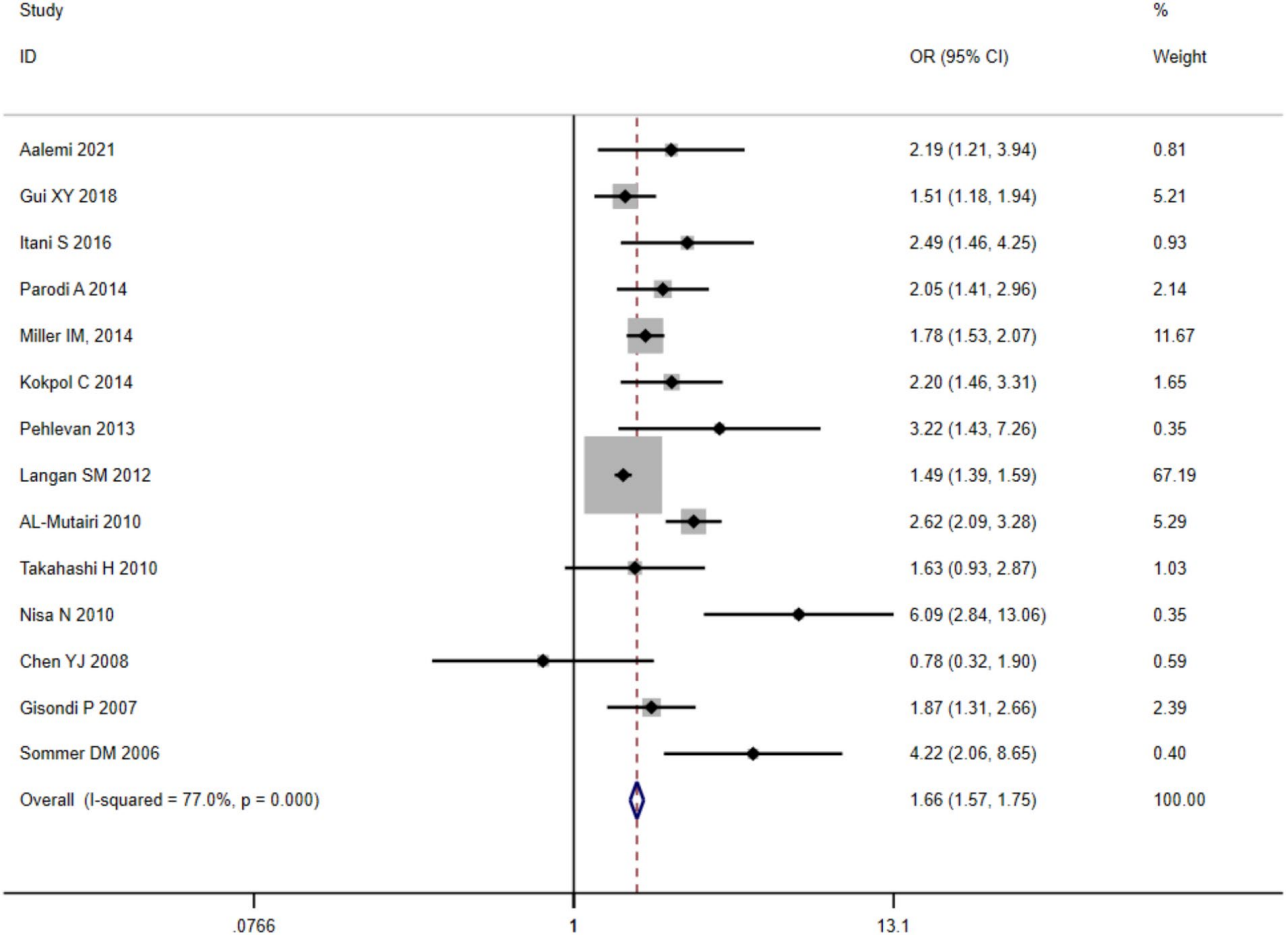
Meta-analysis of the prevalence of metabolic syndrome in the psoriasis patients.

A quantitative synthesis of twelve eligible clinical trials demonstrated substantial between-study heterogeneity ( I2=68% ), necessitating implementation of a DerSimonian-Laird random-effects analytical framework. The pooled prevalence of clinically significant metabolic dysregulation among psoriatic cases reached 26.49% [95% CI (25.61, 27.39%)]. Comparative analysis against matched control populations revealed elevated odds ratios for MetS development in psoriasis cohorts (OR = 1.27, 95%CI (1.21–1.33), *p* < 0.001), with forest plot visualization presented in Figure 2.

#### 3.2.2 Sensitivity analysis and subgroup analysis

**Figure 3:**
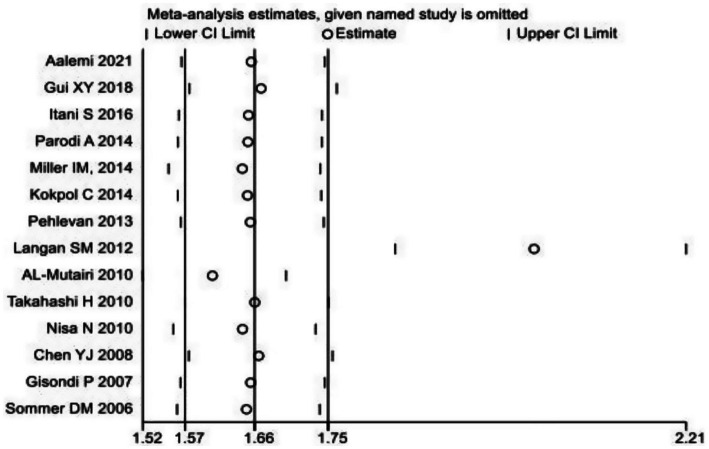
Sensitivity analysis chart of the prevalence of metabolic syndrome in patients with psoriasis.

Iterative validation through a leave-one-out sensitivity framework demonstrated preserved effect magnitude stability when systematically eliminating individual datasets (Figure 3). For the subgroup analysis of MetS, based on PASI scores, the graded MetS risk escalation was higher in cases with severe psoriasis (PASI ≥ 10) versus controls the control group [OR = 2.247, 95% CI (1.267, 3.985), *p* < 0.001], which was statistically significant. Meta-regression confirmed a positive linear trend between PASI increments and MetS incidence.

#### 3.2.3 Publication bias assessment

**Figure 4:**
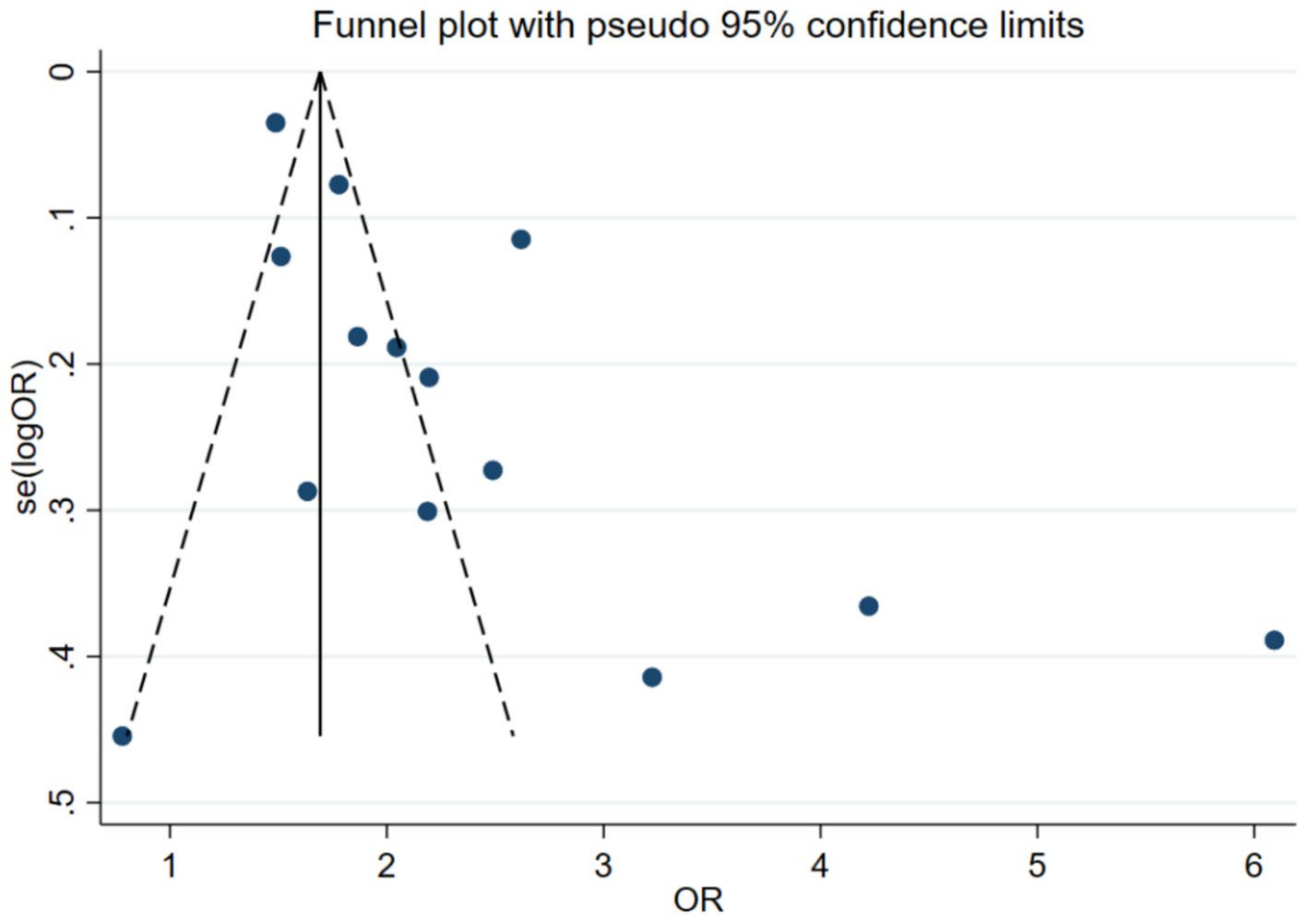
Egger funnel plot of the prevalence of metabolic syndrome in patients with psoriasis.

The publication bias assessment plotted an Egger funnel plot for 14 studies. The result showed that Egger’s regression *p* < 0.05, indicating an asymmetric distribution. The effect sizes observed in small sample studies were different from those in large sample studies, suggesting the possibility of publication bias. The results are shown in Figure 4.

## 4 Discussion

The chronic systemic immune-dysregulation disorder of psoriasis vulgaris impacts between 0.47 and 11.2% of mature populations globally, with pediatric cases reaching 1.37% incidence. The cutaneous condition manifests through sharply defined erythematosquamous keratotic lesions (Koebner phenomenon prevalence: 25–30%), demonstrating recurrent exacerbations interspersed with clinical remission periods. Even with optimized therapeutic interventions, psoriasis can only be containment, not cured. Genetic and environmental factors are thought to be involved in the immunopathogenesis of psoriasis. Psoriasis is associated with multiple comorbidities, including cardiovascular disease, chronic kidney disease, uveitis, mental disorders, and metabolic abnormalities (e.g., obesity, hypertension, dyslipidemia, and both type 1 and type 2 diabetes), all of which can significantly reduce quality of life and life expectancy. Syndrome has been recognized for over eight decades. This grouping of physiological disorders, each of which contributes to the risk of cardiovascular disease, was originally defined as a collection of hypertension, hyperglycemia, and gout. As metabolic syndrome increases the risk of both diabetes and cardiovascular disease, early identification becomes particularly crucial. Clusters of physiological disturbances include glucose intolerance, including type 2 diabetes, impaired glucose tolerance, and abnormal fasting blood glucose levels, insulin dysregulation, abdominal adiposity, dyslipidemia, and hypertension, all of which are well-established risk factors for cardiovascular disease. These conditions frequently co-occur in individuals at rates higher than would be expected by chance. When these diseases are combined, they increase the likelihood of cardiovascular disease. MetS is now commonly defined as a collection of metabolic abnormalities. This group includes abdominal adiposity, insulin resistance, hypertension, and dyslipidemia. Metabolic syndrome encompasses multiple cardiovascular risk factors. Metabolic syndrome is highly correlated with cardiovascular and cerebrovascular diseases, such as myocardial infarction and stroke.

This meta-analysis synthesized evidence from 12 studies involving 9,641 psoriasis patients, of whom 2,554 were diagnosed with concomitant MetS. The included studies include 10 cross-sectional studies (moderate quality, AHRQ score 4–7) and 2 high-quality case–control studies (AHRQ score 8). Our research findings highlight two key observations: 1. Psoriasis as an independent risk factor for MetS: Compared to the non-psoriasis control group, psoriasis patients had a 26.8% increased risk of developing MetS (OR = 1.268, 95% CI: 1.207–1.331). 2. Disease activity regulates the risk of MetS: Severe psoriasis (PASI>10) is associated with a 124.7% increased risk of MetS (OR = 2.247, 95% CI: 1.267–3.985), suggesting a possible dose–response relationship between inflammatory burden and metabolic disorders.

This observation aligns with multiple studies supporting the bidirectional association between psoriasis and MetS. Current evidence identifies MetS as a significant risk factor for psoriasis (OR = 1.52, 95% CI = 1.23–1.88, *p* < 0.05), with abdominal adiposity—a key diagnostic feature of MetS—showing particularly strong correlations with psoriasis severity (7). Clinical investigations highlight promising therapeutic outcomes of apremilast in psoriatic patients with cardiometabolic comorbidities (8), though comorbid MetS independently elevates all-cause mortality in this population (9).

The pathophysiological interplay remains incompletely elucidated, but several mechanistic hypotheses have been proposed: Chronic inflammatory-driven pathway: Proinflammatory cytokines in psoriasis (e.g., TNF-*α*, IL-6, IL-17) systematically induce insulin resistance through suppression of insulin receptor substrates and impairment of glucose uptake in adipose tissue (10). Concurrently, these mediators disrupt lipid homeostasis by upregulating hepatic lipogenesis and inhibiting lipoprotein lipase activity (11). Metabolic reprogramming in keratinocytes: Psoriatic keratinocytes exhibit glucose metabolic rewiring that potentiates inflammatory cascades within MetS-associated microenvironments (12). IL-9-mediated crosstalk: Elevated IL-9 levels in psoriasis patients with MetS comorbidity suggest its potential role as a molecular link between inflammatory and metabolic pathways (13).

Notably, our subgroup analysis revealed a significant correlation between elevated PASI scores and increased incidence of MetS ( p <0.05). This observation aligns with longitudinal evidence demonstrating that systemic anti-psoriasis therapies, particularly IL-17/IL-23 targeted biologics, exert beneficial effects not only on cutaneous manifestations but also improve metabolic parameters including HbA1c reduction and HDL-C elevation^1^. These collective findings support the inflammatory mediation hypothesis, suggesting that attenuation of systemic inflammation may mitigate metabolic comorbidities.

Based on these clinical insights, we recommend structured metabolic surveillance protocols for patients with moderate-to-severe psoriasis, incorporating annual assessments of fasting plasma glucose, comprehensive lipid profiles, and blood pressure monitoring. Cases displaying metabolic irregularities should prompt timely referral to multidisciplinary specialists (endocrinology/cardiology) for optimized comorbidity management. When initiating systemic therapies, clinicians should prioritize therapeutic agents with demonstrated metabolic benefits, particularly in high-risk cohorts.

While maintaining rigorous methodology, this study presents several limitations requiring acknowledgment: (1) Potential geographical and diagnostic heterogeneity: The exclusion of non-Chinese/English literature may introduce cultural bias in MetS diagnostic interpretations. (2) Temporal ambiguity: The predominantly cross-sectional study designs (83% of included studies) preclude definitive causal inference between psoriasis severity and metabolic derangements. (3) Therapeutic confounding: Longitudinal metabolic outcomes might be influenced by concurrent pharmacological interventions not systematically documented across studies.

While the methodology of this meta-analysis was rigorous, several limitations warrant careful consideration. Geographical and Diagnostic Heterogeneity: The exclusion of studies from non-Chinese/English languages could have led to a cultural bias, potentially impacting how MetS is diagnosed and interpreted in different populations. Variations in diagnostic criteria for MetS, along with differing health policies across countries, may affect the consistency of the results. Temporal Ambiguity: The predominance of cross-sectional study designs in the included studies (83%) limits our ability to draw definitive conclusions about the causal relationship between psoriasis severity and metabolic abnormalities. Longitudinal studies are necessary to establish a clearer temporal association and to determine whether the presence of severe psoriasis precedes the development of MetS or if it is a consequence of ongoing inflammatory processes.

Psoriasis and MetS are closely related. Psoriasis is a systemic immune disease, long-term chronic inflammation will affect multiple organ systems of the body, especially organs closely related to metabolism, such as the heart, blood vessels, liver and so on. Inflammatory factors in psoriasis patients, such as TNF-*α*, IL-6, IL-22, etc., in addition to causing skin lesions, also promote the occurrence of metabolic disorders and increase the risk of MetS. Therefore, for psoriasis patients, timely identification and treatment of MetS is of great significance. By effectively controlling various indicators of MetS, such as blood glucose, blood lipids, blood pressure, etc., the risk of cardiovascular disease, diabetes and other serious diseases can be reduced, thereby improving the quality of life and long-term survival expectation of patients. Psoriasis is not only a skin disease, but its effects also affect the whole body and can trigger a range of comorbidities, especially MetS and cardiovascular disease. Timely identification of MetS groups in patients with psoriasis and active intervention and treatment measures can not only reduce the symptoms of psoriasis, but also effectively prevent and control the related complications of MetS. Strengthening research and clinical management of the relationship between psoriasis and MetS is essential to improve the health and quality of life of patients with psoriasis. MetS is frequently observed as a comorbidity of psoriasis. However, recent research has shown that the two conditions not only coexist but also influence and exacerbate each other. With the advent and gradual popularization of biologics, many psoriasis patients have been able to achieve clinical clearance of skin lesions, and the difficulty of treatment has been significantly reduced. The potential harm of MetS as a comorbidity of psoriasis is gradually emerging. Future studies should further explore the possible common immune pathway between psoriasis and MetS, so as to provide a more reliable basis and a more accurate treatment strategy for the clinical treatment of metabolic diseases associated with psoriasis.

Despite the aforementioned limitations, our findings hold significant clinical implications. Firstly, the incidence of metabolic syndrome is elevated in psoriasis patients, particularly among those with higher disease activity. This underscores the importance for clinicians to focus on the early identification and intervention of metabolic syndrome when managing psoriasis. The increased risk of metabolic syndrome in psoriasis patients may be closely linked to their inflammatory levels, suggesting that controlling inflammation could be an essential therapeutic goal to reduce the occurrence of metabolic syndrome. Furthermore, based on our analysis, clinicians should enhance the comprehensive assessment of psoriasis patients, particularly in areas such as weight management, blood glucose monitoring, and lipid profile monitoring, to prevent and detect cardiovascular diseases associated with metabolic syndrome at an early stage. Lastly, as our understanding of the relationship between psoriasis and metabolic syndrome continues to evolve, a multidisciplinary approach integrating dermatology, endocrinology, and nutrition may be beneficial for providing comprehensive treatment and management strategies, thereby improving the overall health of these patients.

## 5 Conclusion

In this study, we found that patients with psoriasis have a significantly increased risk of developing MetS (MetS) and are positively correlated with disease activity. This indicates that psoriasis patients, especially those with more severe conditions, may not only face difficulties with skin symptoms, but may also face higher metabolic health risks. Therefore, in clinical practice, more attention should be paid to the metabolic health of psoriasis patients, regular monitoring should be carried out, and early intervention measures should be taken to prevent the occurrence of MetS. Meanwhile, interdisciplinary collaboration is particularly important, and dermatologists should closely cooperate with departments such as endocrinology and cardiovascular medicine to jointly manage the overall health status of patients.

Future research should further explore the mechanisms between psoriasis and MetS, particularly how the inflammatory response of psoriasis affects metabolic processes. These studies will help deepen our understanding of the relationship between the two and provide a more scientific basis for optimizing treatment strategies and developing personalized management plans.

## Data Availability

The original contributions presented in the study are included in the article/supplementary material, further inquiries can be directed to the corresponding author.
